# Appointment of Professor Dickson

**Published:** 1858-05

**Authors:** 


APPOINTMENT OF PROFESSOR DICKSON.—It affords us much pleasure to announce that, at a meeting of the Trustees of Jefferson Medical College, on the 27th instant, Dr. SAMUEL H. DICKSON, of Charleston, South Carolina, was appointed as the successor to the lamented Mitchell in this Institution. The high reputation of Dr. Dickson, as an able lecturer, a finished writer, and an accomplished gentleman, are too well known to require any comment.

